# Supplementary material for: Quenched Magnon excitations by oxygen sublattice reconstruction in (SrCuO2)n/(SrTiO3)2 superlattices
Source: Sci Rep. 2016 Sep 12;6:32896. doi: 10.1038/srep32896 (PMC5018731; doi:10.1038/srep32896)
Supplement: Supplementary Information [file srep32896-s1.pdf]

# Supplementary inormation to Quenched Magnon excitations by oxygen sublattice reconstruction in $(SrCuO_2)_n/(SrTiO_3)_2$ superlattices

**M. Dantz[1,*], J. Pelliciari[1], D. Samal[2,+], V. Bisogni[1,+], Y. Huang[1], P. Olalde-Velasco[1,+], V. N. Strocov[1], G. Koster[1], and T. Schmitt[1,*]**

[1]Swiss Light Source, Paul Scherrer Institut, CH-5232 Villigen PSI, Switzerland
[2]MESA+ Institute for Nanotechnology, University of Twente, Post Office Box 217, 7500AE Enschede, The Netherlands
[*]Correspondence and should be addressed to M.D. (email: marcus.dantz@psi.ch) or T.S. (email: thorsten.schmitt@psi.ch)
[+]Present adress of D.S.: Institute of Physics, Sachivalaya Marg, Bhubaneswar-751005, India. Present address of V.B.: National Synchrotron Light Source II, Brookhaven National Laboratory, Upton, New York 11973, USA. Present address of P.O.V.: Instituto de Ciencias Nucleares, UNAM, Circuito Exterior, Ciudad Universitaria, 04510 Mexico D.F., Mexico

## ABSTRACT

## Microscopic details

In Fig. S1 we show a TEM image containing the boundary between the $\Pi$ and $\Sigma$ sheet orientations, thus unambiguously showing the existence of both phases in the same sample, as presented in the paper. The experimental settings were same as in Ref.[1]

## Fitting details

In Fig. S2 we show the fit of the orbital excitations of a typical RIXS spectrum. The experimental data is represented by the blue line in the middle section, the fitted line is the broken line in the same section. The top section shows the residuals and the lower section displays the individual Gauss functions used. Peak 1 and 0 correspond to the $d_{z^2}$ orbitals on Cu sites with and without apical oxygen, respectively. Peak 2 represents the $d_{xz}$ and $d_{yz}$ orbitals (degenerate in tetrahedral symmetry) and peak 3 represents the $d_{xy}$ orbital.

The fitted values are $E_{xy} = -1.486 \pm 0.003$eV, $E_{xz/yz} = -1.788 \pm 0.003$ eV and $E_{z^2} = -2.704 \pm 0.003$ eV. The values of $D_t$ and $D_s$ were derived by the equations $\Delta e_g = 4D_s + 5D_t$ and $\Delta t_{2g} = 3D_s - 5D_t$[2], where $\Delta E_g$ and $\Delta t_{2g}$ are the splitting between the $e_g$ and $t_{2g}$ peaks, respectively. In the present case, the former are constituted by the elastic line $(x^2 - y^2)$ and the $z^2$ orbital, while the latter is the splitting between the $xz/yz$ and the $xy$ orbital. The value for $10Dq = 1.486 \pm 0.003$ eV was derived by the splitting between the $x^2 - y^2$ and the $xy$ orbital, in line with previous work[2,3]. However, it should be noted that the $10Dq$ value can also be calculated by the difference of the centroids of the $t_{2g}$ and $e_g$ orbitals, which yields a substantially different value of $10Dq = 0.335 \pm 0.005$ eV. The difference between these values shows that the single ion model is not sufficient to explain the crystal field splittings in these materials and covalency has to be taken into account.

For the fit to the magnetic part of the RIXS spectra as exemplified in Fig. 2b of the main paper, we chose three Gaussian lines to account for contributions of the elastic line (peak A), the single magnon excitation (peak B) and multi-magnon contributions (peak C). Our justification for choosing Gaussian line profiles for both peak A and peak B is that the experimental response function for RIXS in this case is a Gaussian profile and we expect this to be the main contribution to the linewidths of both profiles. For peak C, an asymmetric line shape is expected due to higher order contributions besides the bi-magnon excitations. However, we expect these to be small and have an insignificant impact on the fitted position of the single magnon peak B.

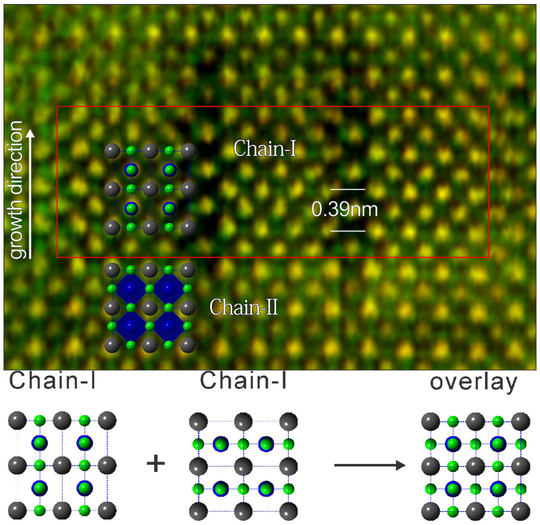


**S 1.** TEM image of phase boundary between both chain types. This is clearly distinguishable from the 2D structure in the thick limit case shown in Ref[1]

## Magnetic excitations

To further elucidate the nature of the magnetic excitations, we measured the 8uc sample also in $\Gamma-$ M direction, see Fig. S4. These measurements were taken in grazing exit geometry and $\pi$ polarisation in order to maximize the cross section of the single magnon excitations and to quench the contribution of multi magnons. We can clearly see that the periodicity of the excitation is as expected for a 2D compound, as a 1D compound would show just a stretched out spinon dispersion.

## References


**1.** Samal, D. *et al.* Experimental Evidence for Oxygen Sublattice Control in Polar Infinite Layer ${\mathrm{SrCuO}}_{2}$. *Phys. Rev. Lett.* **111**, 096102 (2013). URL http://link.aps.org/doi/10.1103/PhysRevLett.111.096102.

**2.** Sala, M. M. *et al.* Energy and symmetry of dd excitations in undoped layered cuprates measured by Cu L3 resonant inelastic x-ray scattering. *New J. Phys.* **13**, 043026 (2011). URL http://iopscience.iop.org/1367-2630/13/4/043026.

**3.** Li, W.-K., Zhou, G.-D. & Mak, T. C. W. *Advanced Structural Inorganic Chemistry* (Oxford Univ Pr, Oxford ; New York, 2008), revised, update. edn.

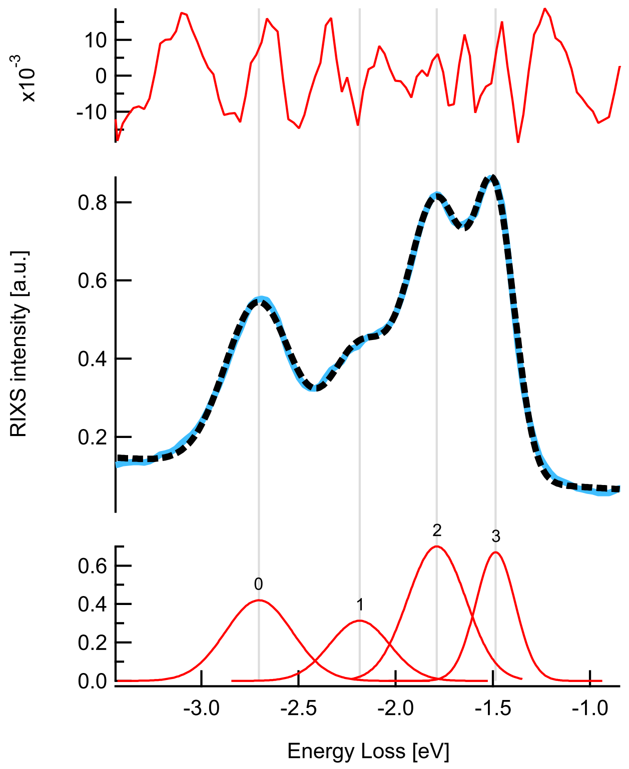


**S 2.** Fitted orbital excitations used to determine 10Dq, Dt and Ds.

.

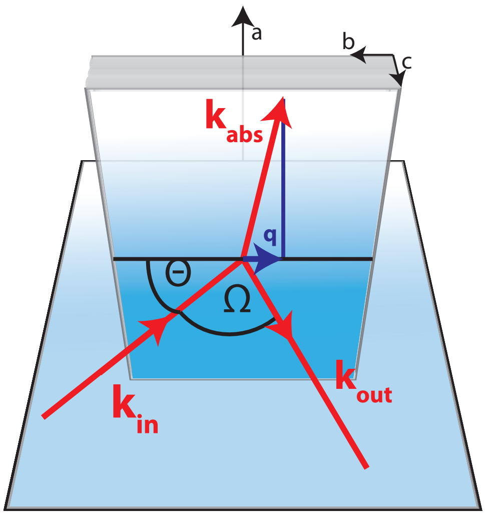


**S 3.** Sketch of the experimental setup and scattering geometry with respect to the sample surface. Ω is the included angle (in the present case, it was fixed at 50°, i.e 180 ° minus the scattering angle of 130°.). Θ is the incidence angle of the beam with respect to the surface and can be used to change the in-plane projection q of the absolute transferred momentum $k_{abs}$ .

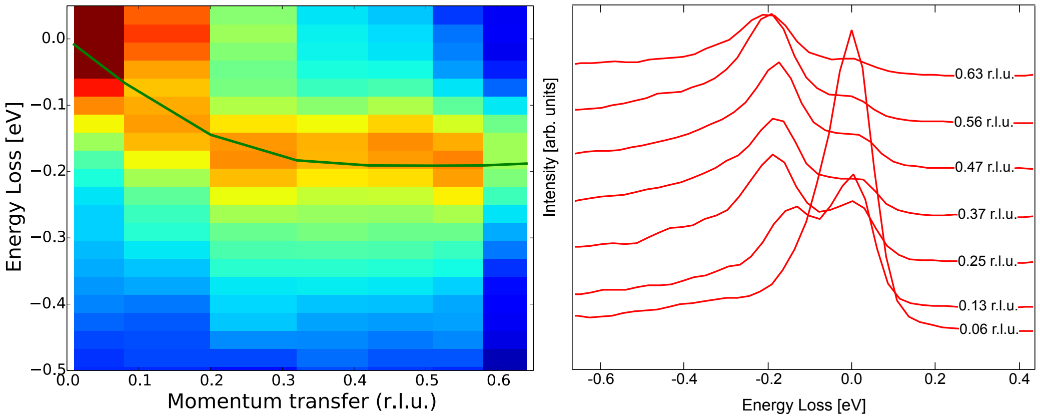


**S 4.** Magnetic excitations for the 8uc sample in Γ - M direction. Right panel: Contour plot of the RIXS data. The line plotted on top represents the Linear spin wave theory given in Eq. 1 in the methods section of the main paper, using the fitted results of J=163meV and Jc=52meV. Right panel: line plots of the same data.
